# Independent Mobility and Social Affordances of Places for Urban Neighborhoods: A Youth-Friendly Perspective

**DOI:** 10.3389/fpsyg.2018.02198

**Published:** 2018-11-13

**Authors:** Frederico Lopes, Rita Cordovil, Carlos Neto

**Affiliations:** ^1^Laboratory of Motor Behavior, Faculdade de Motricidade Humana, Universidade de Lisboa, Lisbon, Portugal; ^2^Centro Interdisciplinar de Performance Humana, Faculdade de Motricidade Humana, Universidade de Lisboa, Lisbon, Portugal

**Keywords:** neighborhood, independent mobility, active travel, social affordances of places, SoftGIS methodology, youth-friendly

## Abstract

Meaning of place is usually approached as slow social cognitive construction. However, grounded on the theory of affordances, it may also stem from direct perception-action processes, which enable the formation of immediate perceived functional, social or symbolic meaning of place (Raymond et al., 2017b). In the present study, affordances of places, which are perceived by a specific perceiver in a specific place, were mapped using a web-map survey. Each place offers opportunities for interaction, behavior, use, feeling or meaning, which is directly perceived and actualized there. This paper aims at identifying the degree of youth-friendliness of urban neighborhoods using the hypothetical intertwined model of independent mobility and actualized affordances (Kyttä, 2004) combined with place use and meaning (Broberg et al., 2013a). SoftGISchildren methodology (Broberg et al., 2013a) was adopted to carry out a cross-sectional research, involving 145 sixth to ninth graders of the Great Lisbon area. SoftGIS integrates Public Participation Geographic Information Systems (PPGIS), enabling collection and place mapping of daily subjective environmental experiences in the physical environment, consequently allowing for individuals to be actively engaged in public processes of participation (Brown and Kyttä, 2014). A place based web survey called “Ideal City: a game of graphic imagination” was adopted. Participants were asked to map their home place, select and mark social, functional, leisure and emotional place transactions, and report actual and ideal mobility to these places. Findings on mobility showed that shorter distances to meaningful affordances of places promote active and independent travel; ideally, youths would like to be more frequently active and more frequently autonomous. As for meaningful places, a total of 1632 affordances were localized, with a higher number on social category. Neighborhood area (500 m around home place) was assessed as youth-friendly, where active and independent travel occurred more frequently, and social affordances were the most expressive type. Relational and affectional experience in the neighborhood places was meaningful for youth. Municipalities should consider these features when planning, designing and managing residential areas aiming for the well-being and health of young citizens; and include youths as specialists of space (*spatialists*) in planning participatory processes (PPGIS).

## Introduction

### Theoretical Mosaic

According to the UNICEF report on the state of world children (United Nations for Children’s Fund, 2012), childhood experience is becoming increasingly urban, since more than one billion children are living in cities and towns. Portugal is no exception, and statistics indicate that the largest population densities of children in Portugal (0–14 years old) is concentrated in urban municipalities, and in the Great Lisbon area this percentage is of 20.5% (Pordata Statistics Portugal, 2011). Independent mobility and person-place relationships are fundamental aspects of youth’s daily lives in the urban environment and are key features that need to be addressed when conceptualizing and planning child and youth-friendly environments, settings, neighborhoods, and structures (Kyttä, 2004; Eisinger, 2012; Whitzman and Mizrachi, 2012; Broberg et al., 2013a; Carroll et al., 2015).

Independent mobility of children or youth in the urban setting can be defined as permission for children to move without adult supervision in their neighborhood and city (Hillman et al., 1990; Tranter, 1994) so that they can explore and learn about the environment at their own rhythm (Björklid and Nordstrom, 2004), toward a progressive and wider freedom of action and movement (Tonucci, 2005). Previous studies have shown Portugal with low levels of children’s independent mobility (Cordovil et al., 2015) and ranked internationally in the 14th place among 16 countries (Bicket, 2013). Moreover, in urban centers, children and youths’ restrictions on autonomous movement in Portugal are particularly augmented (Lopes et al., 2014). Children’s independent mobility is fundamental for children and young people’s health, well-being and overall development. Brown et al. (2008) summarize, from other relevant studies, such benefits: development of motor skills; increase in additional physical activity; influence on cognitive development by helping children to increase their way-finding abilities, and also the development of emotional bonds between children and the natural environment. On the other hand, restrictions on children’s freedom to roam in the environment have been synthesized by Alparone and Pacilli (2012) as hindering the development of motor, spatial, social, and analytical competences; decrease of physical activity and difficulties controlling weight; increase of environmental fears and feelings of loneliness; and weaker sense of community. Moreover, the exclusion of children and of youths from public space constitutes a threat for meaningful participation of young citizens in the matters of the city governance (Tonucci and Rissotto, 2001).

Human movement through the environment becomes more complex throughout development simultaneously that person-place interactions gain further complexity and multidimensionality. Hart and Moore (1973) explain that the child constructs knowledge about the environment through “acting-in-space.” Weston (2010) points out that physical movement through the environment is necessary for learning about it and that young people are physically able to travel independently and are psychologically prone to it. In this sense, the purpose of human mobility is very much related with place experience and vice-versa. Therefore, mobility, namely, independent mobility, is crucial for young people to gain access to diversified socio-physical spaces, where these person-place interactions take place. In this way, on a daily basis, young people roam through different socio-physical environmental settings, where life in places occurs within a complex range of internal and external features, processes, and activities. In this research, the youth-place relationship is addressed through the transactional approach within the field of Environmental Psychology. This theoretical perspective of person–environment phenomena considers them as holistic entities composed of an intrinsic and inter-related assemblage generated through the interplay of people in action, psychological processes, physical environment, and temporal qualities (Altman and Rogoff, 1987; Bonnes and Secchiaroli, 1995; Werner et al., 2002).

According to Gibson (1979/1986) and Heft (2012), an affordance is a psychological relational significant property perceived by the individual when interacting with the socio-physical environment. Thus, it means that when an individual perceives an affordance in a given space through an immediate sensory-action process, a significant feature or cue in the physical landscape specifies a possibility of action according to the individual’s developmental characteristics and the specific feature within such space. Kyttä (2003, 2004) stresses that the environment offers the individual an infinite number of affordances which act as a potential for human multidimensional activity. It is within this range of environmental potential that intentional perception-action cycles take place. For this to happen a matching between the individual’s corporality, expressed by his physical, social and psychological characteristics, skills and necessities, and the material and sociocultural features of the environment has to occur. This process is designated by the author as “actualization.” In this sense, a perceived affordance implies signaling an opportunity in the environment for a transactional experience by a specific actor, while an actualized affordance refers to detecting and establishing a mutual embodied fit with such environmental opportunity or cue. Socio-cultural processes and psychological processes are mutually constitutive through on-going co-emergent human-environment transactions, which means that affordances are nested within the sociocultural tissue and vice-versa as a dynamical system (Heft, 2013). By applying the theory of affordances (Gibson, 1979/1986) in embodied cognition, Raymond et al. (2017a) refer to embodied ecosystems as relational, situational, and dynamical features between humans and the ecosystem. These are self-perpetuated and iteratively reconfigured over time as a consequence of perception-action processes that are actualized through co-constitutive relations between environment, culture, body and mind. These authors also stress that meanings and values of embodied ecosystems extend beyond instrumental functionality to a multiple and diverse array of psychological layers such as symbolic, social, cultural, spatial, etc. Likewise, several authors understand the concept of affordance as multidimensional, which manifests itself through social, emotional, cognitive and cultural properties that children and young people attribute to places, providing them with psychological relevant meaning (Graumann, 2002; Christensen, 2003; Chatterjee, 2005; Min and Lee, 2006; Lim and Barton, 2010).

A more in depth approach to place experience is necessary to understand the complexity of immediate person-place perceptual processes of interaction. Drawing on the work of several scholars, Masterson et al. (2017) have reflected on the influence of sense of place to the research on socio-ecological systems. Such studies refer to sense of place as people’s attachment and meaning to settings in the environment, where the former is an emotional, evaluative type of bond composed by place dependence and place identity; and the latter refers to imagistic descriptive, symbolic and interpretative descriptions of what places are like for individuals. We concur with Raymond et al. (2010) on the idea that the attributes of the socio-physical settings for a specific perceiver and user are intertwined with specific personalized emotions, which are grounded in the personal context of place attachment. Concerning “place meaning,” or how do people create meanings to a place, according to Stedman (2008), settings have the potential for users to create multiple embodied cognitive meanings, referring to the descriptive symbolic meaning that people attribute to a place (Stedman, 2016).

Therefore, in sense of place research it is popular to refer to place attachment and place meaning as slow social constructive processes. Nevertheless, place may also be addressed as a direct perception-action process, grounded on the theory of affordances, which enables the formation of an immediate perceived meaning of place (Raymond et al., 2017b). Meaning of place is perceived directly, in real time, through a mutuality fit between the attributes of the environmental features and those of the perceiver. This perspective is grounded in the transactional perspective of environmental psychology where the person and setting are inseparable, acting as a socio-cultural cognitive embodied ecosystem, where dynamic, multilevel transactions co-emerge within the body, mind, culture and environment (Raymond et al., 2017a). It also means that all the information and meaning necessary for the affordance to be actualized by the perceiver is available in the specific setting to be perceived. In addition, places have intertwined multiple meanings, sensory, inherent, instrumental, socio-cultural and identity-expressive types. Through direct perception and action functional, social or symbolic place meanings are immediately perceived and actualized in the presence of environmental cues and perceptual components that exist in a specific setting. This is opposite to a slower process of creating meaning of places through social construction (Raymond et al., 2017b).

In the present study, the youth in place immediate experience and place meaning is theoretically hinged on actualized affordances of places. These are relational, situational and dynamical properties that temporally co-emerge within an entanglement of relations between the mind, body, culture and the environment, nested in embodied socio-cultural ecosystems (Raymond et al., 2017a). Also, our study is methodologically grounded on a place-based approach designated as SoftGIS, which is grounded on the person–environment transactional approach to place interactions.

*SoftGIS* integrates a wider spectrum of methods and processes addressed as Public Participation Geographic Information Systems (PPGIS). Brown and Reed (2009) refer to PPGIS as the process of using GIS technologies to produce local knowledge toward inclusion and empowerment of marginalized groups. According to Sieber (2006), PPGIS consist of methods that use GIS to foster participatory democracy by widening the spectrum of public involvement in policymaking and contribute for capacity building and social change undertaken by non-governmental organizations, grassroots groups, and community-based organizations.

In SoftGIS methodology, people’s local knowledge of the environment is personal, place-based, action-driven and spatially referenced (Rantanen and Kahila, 2009), stemming from transactional interactions between person–environment. According to Brown and Kyttä (2014), PPGIS methods, which SoftGISchildren integrate, expresses a participatory mapping process that depends on participants’ capacity to recall their experiences in the physical environment, leading to an attribution of meaning and value for specific places. Therefore, SoftGISchildren methodology is theoretically delimited by the transactional approach of people-environment relationships, which is supported by Gibson (1979/1986) concept of affordance.

The seminal work of Kyttä et al. (2012) that focused on mapping children’s meaningful place, and revealing their mobility behaviors and perceived health was groundbreaking. In our view, this study was in fact forthcoming because it revealed that SoftGIS place-based methodology could be used by children and youth to characterize place interaction and child-friendliness of the environment. In our study, we refer to youth’s meaningful places as actualized affordances of places that are categorized according to different types of place transactions. More specifically, by using a computer interface associated with a digital map, participants locate multi-place specific transactions (actions, activities, social behaviors, feelings, and meanings), which take place in the urban environment. This enables each participant to map his or her own specific affordances that are directly perceived and actualized in the different places. These types of interactions were previously determined and grouped by the researchers as social, functional, leisure and emotional categories, similarly to what Kyttä and colleagues did when operationalizing categories of place interaction in the SoftGISchildren study.

SoftGIS*children* methodology, which was designed for research with children and youth about environment quality (Kyttä et al., 2012; Broberg et al., 2013b), is underpinned by environmental child friendliness. This criteria is proposed by Moore (1986) as diversity of environmental resources and access to play and exploration. It was later revised by Kyttä (2003) where an hypothetical model composed by degree of independent mobility and number of actualized affordances defines child-friendliness of a place. In our view, there are more features that strongly contribute for this methodology to be child-centered and child-friendly. The content of the survey, its digital support, its user-friendly characteristics are included in these. Moreover, the communalities between its theoretical nature and the perspective of Sociology of Childhood of the children as active and competent social actors, knowledgeable of their spatial, social and cultural realities (Corsaro, 2011) are not to be dismissed. Other research conducted with SoftGISchildren have proved the online interactive mapping methodology to be very effective in the study of child-place relationships (Broberg et al., 2013b; Bhosale et al., 2015; Stewart et al., 2015).

In this article we underline the neighborhood area as a meaningful environmental setting where youth-place transactions take place. According to Bronfenbrenner (1994), the neighborhood is an important *microsystem* where children and youth’s social interactivity with multi-dimensional features (physical, social, and symbolic) enable or unable progressively complex interaction or activity in the immediate environment. Clark and Uzzell (2002) measured social affordances actualized by adolescents in several settings, and concluded that the neighborhood, school and town center are promoters of both social and retreat behaviors. More recently, Villanueva and colleagues reinforce the need to explore the effect of the neighborhood built environment on child development as fundamental for an urban planning that promotes a healthy development (Villanueva et al., 2016). Additionally, in New Zealand an ongoing research project, using SoftGIS methodology, is addressing the study of the associations between neighborhood built environments and children’s independent mobility, active travel, physical activity and place interactions (Oliver et al., 2016). Therefore, the neighborhood is a pivotal setting to study children’s and youth’s independent mobility (Stewart et al., 2015; Tranter, 2015), which is a crucial aspect to promote health and healthy lifestyles. For the present study, neighborhood area was defined as a 500 m buffer around each participant’s home. On this, Vanloon (2011) points out that the most popular approach to define a child’s neighborhood is to define a circular buffer around home or school with size and shape informed by theory and/or empirical data. Similar studies, adopting the same methodology, used a 500 m residential buffer (Kyttä et al., 2012; Broberg and Sarjala, 2015).

Neighborhoods are conceived as embodied socio-cultural ecosystems which are relational, situational and dynamical, the same way affordances are (Raymond et al., 2017a). The neighborhood is composed by a diversified range of behavioral-settings that are characterized by a dynamical interdependence between subject and a specific socio, cultural and physical setting (Barker, 1968; Wicker, 2002). Children’s meaningful places result through children’s social participation, mediated by shared intersubjective collective actions, supported by affordances, in specific location, over a period of time (Heft, 2018). Thus, neighborhood places for children and youth are embodied ecosystems that are nested in specific affordances, which are in hand nested to specific places.

To assess youth-friendliness degree of the urban neighborhoods, we draw on two previous interrelated research works. Herein, Kyttä (2004) conducted a cross cultural study about assessing the child-friendliness of Finnish and Byelorussian urban neighborhoods, developing an hypothetical model comprised by the analysis of children’s independent mobility and number of actualized affordances, which results in four possible types of settings. The *Bullerby* type is constituted by opportunities for independent mobility and diversity of actualized affordances, where children and young people establish a positive interrelated cycle between mobility licenses and the actualization of affordances leading to a continuous free roaming and perceived, utilized and shaping of affordances (most child-friendly). The *Glasshouse* type is constituted by the same number of actualized affordances as the previous one. This means that the spatial environment of the neighborhood is rich in transactional place experiences for children and youths to engage. However, due to independent mobility restrictions, the promoted and free fields of action are limited. The *Wasteland* type is composed by opportunities for independent mobility in the neighborhood and a low number of actualized affordances due to the monomorphic properties of the environment. The *Cell* type is any environment, or setting where children are trapped inside, constituted by restrictions to free roaming in the neighborhood, preventing children and youths of perceiving potential affordances. In the other aforementioned research (Broberg et al., 2013a), focus was set on the influence of built environment objectively measured features on environmental child friendliness, adopting SoftGISchildren methodology. It was concluded that independent mobility and richness of affordances are interconnected concepts; child and youth friendliness should be analyzed not as an attribute of the whole environmental context, but as place specific concept through place experience and place meaning.

### Research Goal and Niche

The overarching goal of the present study was to identify youth-friendliness degree of the urban neighborhood through an assemblage of indicators on mobility behavioral patterns and affordances of places. These are conceptualized as immediate different types of place experiences and of place meanings. The focus is set on child-friendliness of places instead of an approach focused on child-friendliness of the environment as a whole. Affordances are mapped through the use of a youth-friendly participatory place–based survey (SoftGIS), which provides an individualized approach of person in place relations. This represents a leap from an immediate sensory experience that takes place when the person is physically and spatially in contact with the immediate environment, because the person is actually in front of a computer screen looking at the daily environment and locating affordances of places through the use of web-map survey. Such assumption poses a risk to the original conception of the “affordance” as a functional significant environmental property detected by the individual in the immediate environment which provides an opportunity for action (Gibson, 1979/1986; Heft, 1988; Gibson and Pick, 2000). In one hand, it is true that the SoftGISchildren survey does not allow for participants to use their sensory perceptual devices in the immediate socio-physical environment, and hence does not allow for the significant relational features between the perceiver and the immediate physical environment to be detected. In the other hand, if subjects are in fact able to digitally detect a mutuality fit between themselves and the displayed environment, by detecting web-localized meaningful spaces, which are then perceived as multi-dimensional affordances of places, they are then reporting place experience as a fast direct perception-action process, grounded on the theory of affordances, which enables the formation of an immediate perceived meaning of place (Raymond et al., 2017b). Moreover, it implies that perceived place meanings are being actualized in the presence of environmental cues and perceptual components that exist in a specific socio-physical setting through a digital interface, and, simultaneously, that meaning of place can be detected immediately and directly through a place-based survey. Within this perspective SoftGIS surveys enable participants to recall their environmental place experiences and map them as actualized affordances of places.

Moreover, this research addresses the notion of actual versus ideal mobility in the urban environment according to youth’s perspectives. To the best of our knowledge, this is the first study using SoftGIS methodology that provides such an approach. Additionally, place based interactions and meanings, and actual and ideal mobility to meaningful places are pivotal to address children’s right to the city, in terms of place attachment, place meaning, social activity, play, leisure, and participation.

According to several scholars, the study of mobility and person-place relationships in the socio-physical environment as intertwined topics offers a valuable perspective of social meaningful research for the development of healthier and happier communities (Kyttä et al., 2015; Laatikainen et al., 2017). As an important social outcome, the present study aims to reinforce the need to include youth’s perspectives when designing urban neighborhoods suitable for their developmental needs and rights as citizens; and, simultaneously, as a means to promote well-being and the development of healthier communities through SoftGIS methodology.

## Materials and Methods

### Participants and Geographical Context

A total of 145 sixth to ninth graders, aged 11–17 years old (*M*_age_ = 12.41; *SD* = 1.43; girls = 48.3%), from three schools located in the Great Lisbon area, were included as participants of this research (“L” group). All places that were marked outside each participant’s municipality were excluded.

The characterization of our sample was devised through consultation of the Statistics Portugal web site (INE) and of other official web sites of the municipalities and parishes where the three schools are located. Although each school represents different geographical locations, west, coastal and northeast of the Great Lisbon area, as a whole these three areas share a similar degree of urbanization and cultural trends and are representative of the social and built tissue that characterizes the urban environment of the Great Lisbon Area in its diversity and complexity. The school (*n*_participants_ = 40; *M*_age_ = 13.03; *SD* = 1.73) in the west area is located in the parish of Belém, in Lisbon municipality. This parish has an area of 10.43 km^2^ with 16551 inhabitants. The school (*n*_participants_ = 52; *M*_age_ = 11.73; *SD* = 0.93) in the coastal area is located in the parish of Paço de Arcos, in Oeiras municipality. The municipality of Oeiras faces the river/sea front and it is spread over an area of 45.72 km^2^, with a total of 172120 inhabitants. The school (*n*_participants_ = 53; *M*_age_ = 12.57; *SD* = 1.32) in the northeast is located at the parish of Parque das Nações, in Lisbon municipality. This parish spreads over an area of 5.44 km^2^ with a total of 21025 residents.

In order to conduct this study, Ethics approval was obtained from the Portuguese Data Protection Authority and from the Ethics Committee of Faculdade de Motricidade Humana, Universidade de Lisboa. Authorization was also granted from the General Department of Education-Portuguese Education and Science Ministry. Moreover, parental and children’s consent was obtained.

### Methods and Data Collection

*“SoftGISchildren”* methodology was adopted in a cross-sectional research. Data collection occurred during school hours in computer equipped classrooms with internet connection. Each session took between 45 min to 1 h and 8–20 children, filled in the SoftGIS survey at the same time. The researcher was always present in each data collection moment. A research assistant accompanied the researcher when there were more than 10 children. Before participants started to complete the survey, the researcher provided a brief explanation of the questionnaire and of the place mapping procedures. Additionally, the researcher made clear that when participants were selecting affordances to be localized in the web-map, each listed affordance should be interpreted as *“a place where I…”*. For example, the affordance *“being with friends”* should be interpreted as *“a place where I am with friends.”* Moreover, the researcher and the research assistant helped those children who found difficulties completing the web-questionnaire, namely, by clarifying questions and helping them to locate meaningful places. Data collection occurred between October of 2013 and February of 2015.

#### SoftGISchildren Survey “Cidade Ideal: Um Jogo de Imaginação Gráfica!”

The “Cidade Ideal: um jogo de imaginação gráfica!” (Ideal City: a game of graphic imagination!) survey’s content, including the grouping of affordances in each expressional category, was inspired in the work of Kyttä et al. (2012) about the use of SoftGIS to reveal children’s behavioral patterns and meaningful places. The questionnaire was translated to Portuguese and its content was revised, reshaped and renamed according previous studies conducted in Portugal and abroad on children’s independent mobility and place interactions (Arez and Neto, 1999; Lopes et al., 2011; Cordovil et al., 2012a,b; Shaw et al., 2012). SoftGISchildren “Ideal City: a game of graphic imagination!” web-map survey was structured in nine pages. Participants were asked to map their home place, select and mark meaningful place transactions, under each of the four available expressional categories (social, functional, leisure, and emotional), provided by Kyttä et al. (2012). In the present study, these meaningful places were designated “*multidimensional affordances of places.”* An innovative aspect of the “Ideal City” digital survey was the inclusion of questions related to what type of mobility participants would like to have when traveling to meaningful places in their ideal city.^1^ To the best of our knowledge, it is the first time in a research work using SoftGISchildren methods, that the web-map questionnaire has been used to analyse children’s actual mobility vs. ideal mobility to meaningful places.

#### List of “Affordances of Places”

**FIGURE 1:**
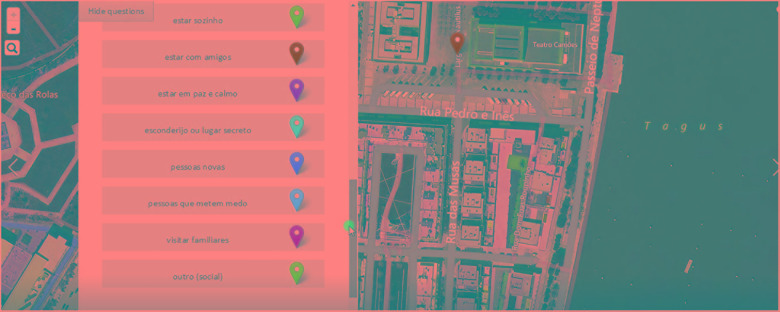
Example of affordances of places available for selection using “Ideal City” SoftGISchildren survey. The figure describes examples of social place activities, experiences, places or meanings which become social affordances of places if selected and located in the web-map by participants. From top to bottom, these are being alone, being with friends, being in peace and quiet, hiding or secret place, new people, scary people, visit relatives, other (social).

The list of social, functional, leisure, and emotional affordances of places (i.e., locations that youths reported to afford place-based specific social, functional, leisure, and emotional activities), is presented in Tables 1–4, accordingly. In Figure 1, an example of the selection panel of affordances of places available for the users in the “Ideal City” SoftGISchildren survey is available.

**Table 1:** Taxonomy for expressional category of social affordances of places.

Social affordance sub-sets	Criteria (place interactions where)	Affordances of places
Privacy	It is mainly valued being alone and free from the public eye	Nobody is watching Being alone Hiding or secret place
Relational	It is mainly valued being connected with others	Being with adults Being with animals Being with friends New people Visit relatives
License	It is/isn’t requested the permit from an authority to actualize it	Forbidden place Allowed place
Affectivity	It is mainly valued the social experience and consequent emotional outcome	Being mistreated Scary people Being myself Being in peace and quiet Place of arguing


**Table 2:** Taxonomy for expressional category of functional affordances of places.

Functional affordance sub-sets	Criteria (place interactions where)	Affordances of places
Locomotor play	Action is mainly focused on *Locomotor Play* *“movement in any or every direction for its own sake”* (Hughes, 2006)	Playing hide and catch Jumping Running Climbing Walking Swimming
Object play	Action is mainly focused on *Object Play “play which uses infinite and interesting sequences of hand-eye manipulations and movements”* (Hughes, 2006)	Skating Riding a bike Playing ball games Going on the swings
Mastery play	Action is mainly focused on *Mastery Play “control of the physical and affective ingredients of the environments”* (Hughes, 2006)	Water playing Playing with sand or earth Building things


**Table 3:** Taxonomy for expressional category of leisure affordances of places.

Leisure affordance sub-sets	Criteria (place interactions where)	Affordances of places
Cultural activities	Activities are mainly focused on engaging participants with ideas, customs and social behavior of societies	Cinema Museums or/and exhibition Library Show/concert/disco Musical events
Outdoor activities	Activities are mainly focused on the exploration of the outdoor environment	Adventuring Parks Gardens
Recreational activities	Activities are done for enjoyment	Playing Having fun Nothing to do Hobbies Hanging out Going out after dark Listening to music Leisure time center
Screen activities	Activities are mainly focused on the use of electronic devices	Playing computer/PlayStation/ electronic games
Physical and sport activities	Activities are mainly focused on physical activity and practice of sports	Sports (football, swimming or other) Dancing (hip-hop, ballet, or other)
Consumption activities	Activities are mainly focused on the use of goods and resources	Shopping Going out for a meal


**Table 4:** Taxonomy for expressional category of emotional affordances of places.

Emotional affordance sub-sets	Criteria (place interactions where)	Affordances of places
Feelings	The experience of feelings is underlined	Fun Calm Good place to be Boring
Aesthetic	It is mainly valued the aesthetical experience	Pretty Ugly Untidy Tidy
Safety	It is mainly valued safety issues	Dangerous Unsafe Safe
Stressors	The experience of environmental stressors, such as light, noise, etc., is underlined	Dirty Clean Polluted Unpolluted Quiet Noisy Dark


#### Taxonomy for Social, Functional, Leisure, and Emotional Expressional Categories of Affordances of Places

For each expressional category of interaction, it was made available an extensive number of affordances of places for participants to select and localize on the web-map (see Tables 1–4 for the full list of affordances of places). Therefore, the main goal of the researchers when creating a hypothetical taxonomy for different expressional categories of affordances (social, functional, leisure, and emotional) was to provide a more analytical perspective of place experiences chosen by participants; and to display an extra qualitative layer of analysis to the current SoftGIS study, namely, in terms of characterizing place use and or place meaning. Also, grouping affordances into sub-sets within the same category of interaction provides an innovative research analysis framework in place based person–environment transactional studies. No statistical procedure was adopted to group affordances of each expressional category in different sub-sets. This procedure was solely empirically driven. For each *“affordance sub-set”* one criteria was devised and preceded by the phrase *“place interactions where”* (see Tables 1–4). Criteria for social, leisure, and emotional expressional categories of affordances was conceptualized based upon definitions of terminologies (Oxford University, 2015) used to name the sub-sets of affordances. As for the criteria for the expressional category of functional affordances it was used a *Playworker’s Taxonomy of Play Types* (Hughes, 2006) due to the behavioral dimension of each affordance in this specific category.^2^

#### Research Analysis Framework

A specific research framework was composed in three inter-connected “layers,” *mobility, affordances of places, neighborhood area*, aiming to provide one of possible transactional landscape on the youth-urban environment relationship in the Great Lisbon Area.

Direct data was imported from the SoftGIS*children* application *“Cidade Ideal: Um jogo de imaginação gráfica!”* to *QGIS 2.8.3.-Wien* and to *IBM SPSS Statistics 22* software. Statistical analysis was performed using SPSS and Excel software on two distinct datasheets, one focusing on the participants general characterization and questions on mobility in between home and school; and another one focusing on the selected meaningful places/affordances and mobility issues. Linear distances between home/school and meaningful places were calculated using *QGIS* software. This same software was used to generate map pictures of meaningful affordances of places. Indirect data provided by place-affordance classification according sub-sets on expressional categories of affordances of places were also added to the meaningful places’ SPSS data sheet and imported to the QGIS software.

#### Research Variables

*Age* (3 age groups: “11–12 years old”; “13–14 years old” and “15–17 years old”) and *Gender* (girls and boys) were operationalized as categorical variables in the SPSS data sheet.

*School-home distance* was calculated by determining mean linear distance (in meters and converted afterwards to kilometers) between participants’ homes and the school which was attended by them.

*Actual and ideal school-home mobility* were determined by analyzing children’s single choice answers on *travel mode* and *travel accompaniment*. For the former (descriptive purpose), *active travel*, when choice included “on foot,” “by bicycle,” or “by other (skate, scooter, roller-skate, etc.); *motorized travel*, when choice included “by car”; *hybrid travel*, when choice included “by bus/by public transport.” When considering actual mobility vs. ideal mobility, travel mode was operationalized as a dichotomous variable, *active travel* and *non-active travel* (*motorized* or *hybrid*). As for travel accompaniment, it was operationalized as *independent travel*, when choice included “alone” or “with other children”; and *non-Independent travel*- when choice included “with adults” or “with adults and other children.”

*Meaningful affordances of places* were operationalized as actualized affordances located in the web-map environment considering four predetermined expressional categories, social, functional, leisure and emotional, which were selected by participants when completing the web-map questionnaire. More specifically they were designated as *social, functional, leisure, and emotional affordances of places* considering each designated expressional category of place interaction. Subsequently, all selected affordances within its expressional category were classified as nominal variables according the sub-sets proposed in each of those expressional categories. This data was then introduced in the SPSS data sheet and in the QGIS software.

*Distance between home and meaningful places* was operationalized as “*territorial distance”* and calculated using the QGIS software by determining mean linear distance (in meters and converted afterwards to kilometers) between participants’ homes and meaningful places where affordances were actualized. Subsequently, this new variable was imported to the SPSS data sheet.

*Neighborhood area was defined by a* circular buffer of 500 m around each participant’s home. This variable was created to classify affordances of places in terms of being located in or out of the neighborhood area. All affordances of places located within 500 m of the respective home place (linear distance) were classified as being “within the neighborhood,” while others marked over 500 m were classified as “beyond the neighborhood.”

*Mobility to meaningful places* other than school was determined by analyzing participants’ multiple choice answers on travel mode and travel accompaniment, after locating each meaningful affordance in the web-map. This means that when analyzing mobility to meaningful places, focus is not on the actual participant but on the place determined via the location of an affordance, and its multiple possibilities of being traveled to regarding travel mode and travel accompaniment. Travel mode was operationalized as three variables (not mutually exclusive). *Active travel* if choice included “on foot,” “by bicycle,” or “by other (skate, scooter, roller-skate, etc); *motorized travel* if choice included “by car”; and *hybrid travel* if choice included “by bus/by public transport.” Travel accompaniment was operationalized as two variables (not mutually exclusive). *Independent travel* if choice selection was “alone” or “with other children” and *non-independent travel* if it included “with adults” or “with adults and other children” (presence of both simultaneously and therefore not autonomous). In the SPSS data sheet, each of these variables was coded individually.

#### Research Topics, Research Questions, and Statistical Procedures

##### Mobility

Frequency analysis and Chi-square tests were performed to investigate whether participants’ age groups or gender were associated with actual mobility and travel accompaniment in the school-home journey, and with actual mobility and travel accompaniment to meaningful places. A univariate analysis of variance was performed to determine if actual mobility (active, motorized, or hybrid) was related with school-home distance in the school-home journey. Concerning the home-school travel mode, participants’ choice was attributed in three mutually exclusive available possibilities (active, motorized, and hybrid). The dependent variable was distance and the independent variable was each travel mode. Hence, statistically, three separate sub-groups (one according each travel mode) were constituted from the whole research group (L). Independent samples *t-*tests were used to determine if travel accompaniment was related with traveling distance in the school-home journey and if actual mobility (active, non-active travel) was related with traveling distance to meaningful places. As for home-school accompaniment, participants’ choice was attributed in two mutually exclusive available possibilities (independent travel and non-independent travel). The dependent variable was distance and the independent variable was each travel type of accompaniment. Hence, statistically, two separate sub-groups (one according each travel accompaniment) were constituted from the whole research group (L). The differences between actual and ideal school-home mobility and actual and ideal mobility to meaningful affordances of places were investigated using *McNemar* tests.

##### Affordances of places

Frequency analysis was performed to investigate: (i) which affordances of places were most actualized, (ii) how frequent was the actualization of each expressional category of affordances and (iii) which sub-sets of affordances of places were most actualized.

##### Neighborhood area

Frequency analysis was performed to investigate variations on (i) actual mobility to meaningful places and (ii) the actualization of different categories of affordances of places when roaming in the neighborhood area.

## Results

### Mobility

#### Age Groups and Actual School-Home Mobility

A significant association was found between age groups and travel modes from home to school (Fisher’s test, *p* < 0.001). Considering active travel mode (walking or cycling), this value rises as participants’ age increases (23.5, 25, and 46.2%, for 11–12 years old, 13–14 years old and 15–17 years old, respectively). As for hybrid travel (public transportation), this value also rises with participants’ age (9.9, 45, and 53.8%, according each of the previous mentioned age groups). Regarding motorized travel, conversely, and as expected, these values decrease as participants’ age increases (66.7%; 29.5% and 0% from younger to older age groups). As for independent travel in the school-home journey, it was found to significantly increase as participants’ age rises, with values of 29.3, 65.1, and 84.6%, in the 11–12, 13–14, and 15–17 years old groups, respectively [χ^2^(2) = 23.39, *p* < 0.001].

#### Gender and Actual School-Home Mobility

Results indicate that there was no statistical significance relationship between gender and children’s actual school-home travel mode (*p* > 0.05) and travel accompaniment (*p* > 0.05).

#### School-Home Distance and Actual School-Home Mobility

It was found a significant effect of school distance on children’s mode of travel from school to home in “L” research group [Welch’s *F*(2,72.92) = 8.65, *p* < 0.001]. *Post hoc* comparisons indicated that: (i) significant differences were found comparing mean distance traveled by the group of youths using active travel mode with mean distance traveled by the group using hybrid travel mode; (ii) significant differences were found comparing mean distance traveled by the group of youths using active travel mode with mean distance traveled by the group of youths using motorized travel mode; (iii) no significant differences were found comparing mean distances traveled by the group of youths using hybrid travel mode with mean distance traveled by the group of youths using motorized travel mode. Hence, the mean distance traveled by youths adopting active travel mode is significantly smaller (*M* = 1125 m, *SD* = 2113) than mean distance traveled by youths using hybrid travel mode (*M* = 3174 m, *SD* = 2450) (*p* = 0.001); and the mean distance traveled by youths adopting active travel mode (*M* = 1125 m, *SD* = 2113) is also significant smaller than mean distance traveled by youths using motorized travel mode (*M* = 2578 m, *SD* = 2062) (*p* = 0.003). These results indicate that participants’ active travel from school to home takes place if school-home mean distance is around 1.1 km. This is particularly relevant when taking in consideration that mean distance between school and home was found to be 2.3 km. In terms of school-home travel accompaniment, no significant differences were found comparing mean distance traveled by the group of youths who traveled independently with mean distance traveled by the group of youths who traveled non-independently.

#### Actual and Ideal School-Home Mobility

The *McNemar* test showed significant differences (*p* < 0.001) when comparing children’s actual and ideal school-home mobility. Only a small percentage of children in the “L” research group traveled actively from home to school (27%), and nearly half of the children reported traveling autonomously in this journey (44.3%). Contrary, and ideally, the vast majority of children in “L” research group would like to be more active, decrease car transportation, and be more autonomous in the school-home journey (Table 5).

**Table 5:** Actual and Ideal school-home mobility in L group.

		Real mobility (%)	Ideal mobility (%)	Statistical significance
Travel mode	Active travel	27.0	66.0	*p* < 0.001
	Non-Active travel	73.0	34.0	
Travel accompaniment	Independent travel	44.3	85.0	*p* < 0.001
	Non-Independent travel	55.7	15.0	


#### Age Groups and Actual Mobility to Meaningful Affordances of Places

**FIGURE 2:**
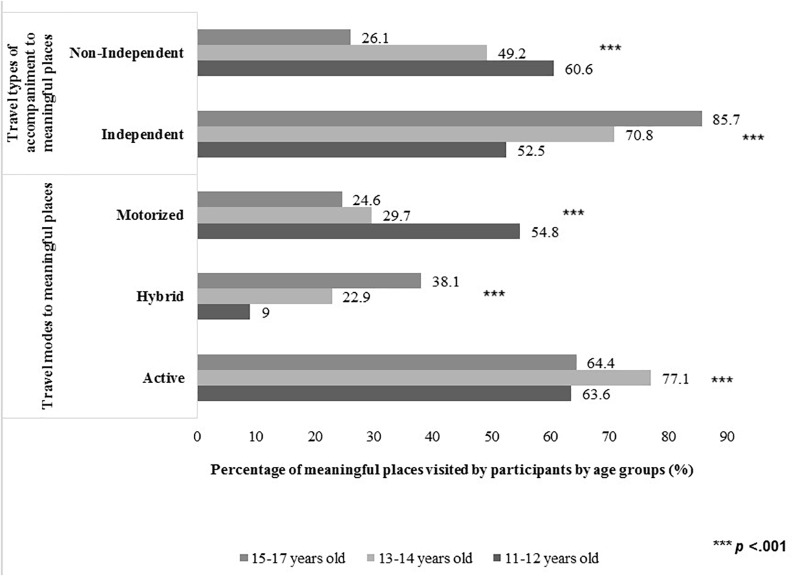
Actual mobility to meaningful affordances of places according age in L group. The answers on travel modes and travel types of accompaniment are multiple choice and therefore they are not mutually exclusive.

In Figure 2, results on the interplay of age groups and mobility to places where children actualized affordances are fully presented. Our findings revealed that in all age groups active travel was the most frequently mode used to access meaningful places. Children aged 13–14 years old more frequently used active travel mode to meaningful places when compared with participants from the other two age groups [χ^2^(2) = 24.18, *p* < 0.001]. Children aged 15–17 years old significantly used more frequently hybrid travel mode to meaningful places when compared with participants from the other two age groups [χ^2^(2) = 80.75, *p* < 0.001]. Children aged 11–12 years old used motorized travel more often than children from the other two groups to access meaningful places [χ^2^(2) = 88.01, *p* < 0.001]. As for the relationship between age and travel type of accompaniment to meaningful places, traveling autonomously or in the company of other children (independent travel) was the most frequently used for the 13–14 years old and 15–17 years old age group; whereas for the youngest age group of children (11–12 years old), more often they traveled to meaningful places in the company of adults. Also, it were found statistical significant differences between the three age groups in terms of independent travel [χ^2^(2) = 71.69, *p* < 0.001] and non-independent travel [χ^2^(2) = 60.00, *p* < 0.001] to meaningful places. Particularly, older children travel autonomously more frequently to meaningful places and less often in company of adults than younger children do.

#### Gender and Actual Mobility to Meaningful Affordances of Places

Gender was only found to be an influential variable when considering the option of hybrid travel to meaningful places, but not very significantly. More frequently girls (18.1%) used public transportation than boys (14.1%) when traveling to meaningful places [χ^2^(1) = 3.83, *p* = 0.050]. In terms of travel accompaniment to meaningful places, significant differences were found between girls and boys. Herein, more frequently boys (65.5%) than girls (59.9%) traveled independently (by themselves or in company of friends) to these places [χ^2^(1) = 4.69, *p* = 0.030]. Conversely, more frequently girls (60.4%) than boys (48.1%) traveled accompanied by adults (non-independently) to meaningful places [χ^2^(1) = 22.27, *p* < 0.001].

#### Distance and Actual Mobility to Meaningful Affordances of Places

Our results showed an interplay between distance and use of different travel modes to meaningful places. Significant differences were found comparing mean distances traveled when adopting active travel and mean distances traveled when not using active travel [*t*(529) = 9.82, *p* < 0.001]. Mean distance for active travel was of 1.3 km (*M* = 1.337, *SD* = 1.806), whereas for other modes of travel this value increased to 3 km (*M* = 3.018, *SD* = 3.255).

#### Actual and Ideal Mobility to Meaningful Affordances of Places

When comparing real and ideal travel modes used by participants when visiting meaningful places to actualize affordances, the McNemar’s test showed significant differences on active travel with an increase from 68.7 to 79%, accordingly (*p* < 0.001). The opposite trend was found in motorized travel mode, diminishing from 43.9 to 27.7%, respectively. As for hybrid travel, no significant differences were found on the real and ideal settings. Considering travel type of accompaniment, a significant increase on real to ideal independent travel was found, 61.9–83.2%, respectively (*p* < 0.001); together with a significant decrease on non-independent travel on both settings, with the values of 54.6 and 31.8% (*p* < 0.001).

### Affordances of Places

#### Actualization of Affordances of Places

**FIGURE 3:**
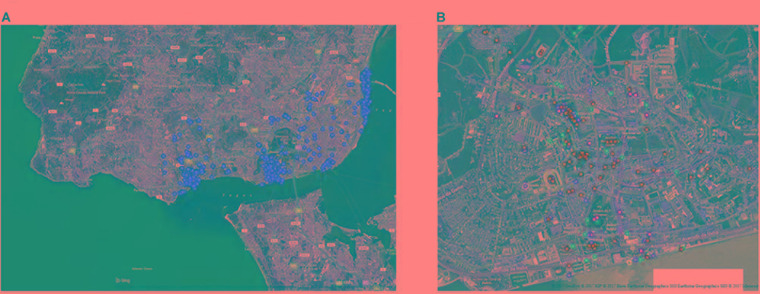
Total number of affordances of places localized by participants in L group **(A)**. Example of social (green dots), functional (orange dots), leisure (yellow dots), and emotional (blue dots) expressional categories of affordances of places localized by participants from LW group **(B)**.

A total of 1777 places were identified, 145 of them were home places corresponding to the total number of this research participants’ and 1632 corresponding to affordances of places distributed in four expressional categories (social, functional, leisure, and emotional). A mean number of 12.26 affordances of places were actualized by each participant. In L group, the highest frequency of actualized affordances within the four conceptualized expressional categories was “social” (35.4%), followed by “leisure,” “functional,” and “emotional” types, with 27.7, 21.6, and 15.3%, respectively. This same decreasing trend in the actualized affordances of places was found for both boys and girls. Figure 3 shows the total number of affordances of places identified by the participants in this study (left panel), and an example of the different expressional categories of affordances of places identified in a particular location (right panel).

**FIGURE 4:**
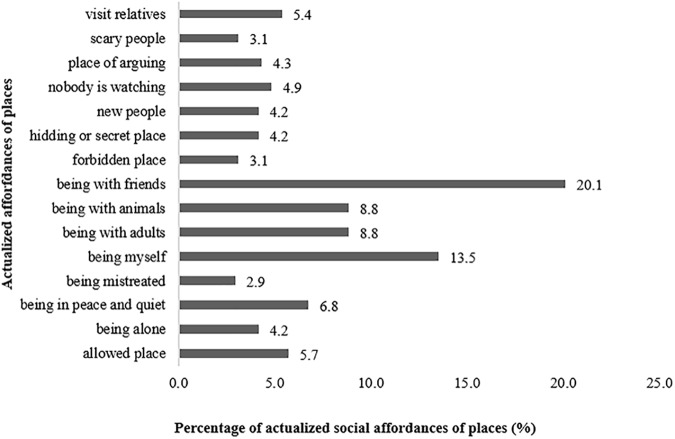
Percentage of social affordances of places actualized by participants in L group.

Within social expressional category of affordances, those most actualized by participants in L group were “being with friends” (20.1%), “being myself” (13.5%), “being with adults” (8.8%), “being with animals “ (8.8%) and “being in peace and quiet” (6.8%), as described in Figure 4. As for social sub-sets, those with higher expression were “relational” and “affectivity” with 47.3% and 30.7%, respectively.

**FIGURE 5:**
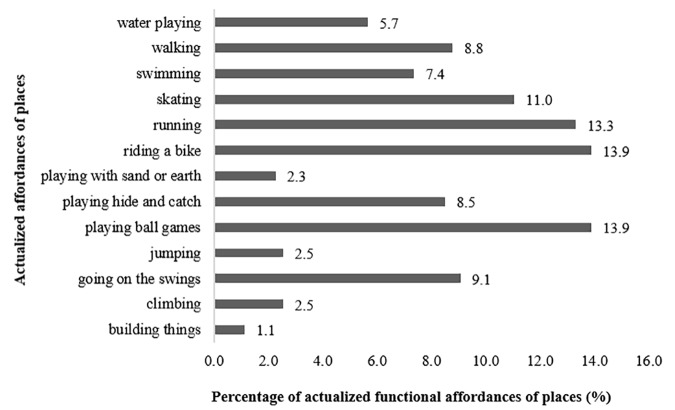
Percentage of functional affordances of places actualized by participants in L group.

In what concerns functional expressional category, most actualized affordances were “playing ball games” (13.9%), “riding a bike” (13.9%), “running” (13.3%) and “skating” (11%), as described in Figure 5. The most expressive functional sub-sets were “object play” (47.9%) and “locomotor play” (43.1%).

**FIGURE 6:**
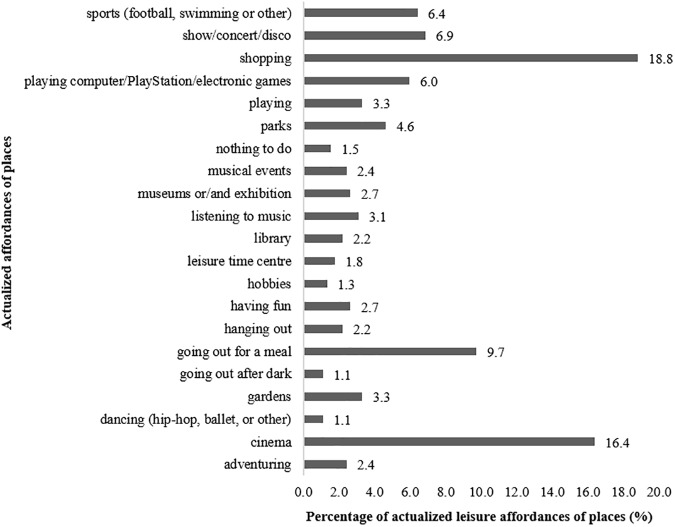
Percentage of leisure affordances of places actualized by participants in L group.

Considering leisure expressional category, most actualized affordances were “shopping” (18.8%), “cinema” (16.4%), “going out for a meal” (9.7%), “show/concert/disco” (6.9%), and “sports” (6.4%) as described in Figure 6. The most expressive leisure sub-sets were “cultural activities” and “consumption activities,” with 30.5 and 28.5%, accordingly.

**FIGURE 7:**
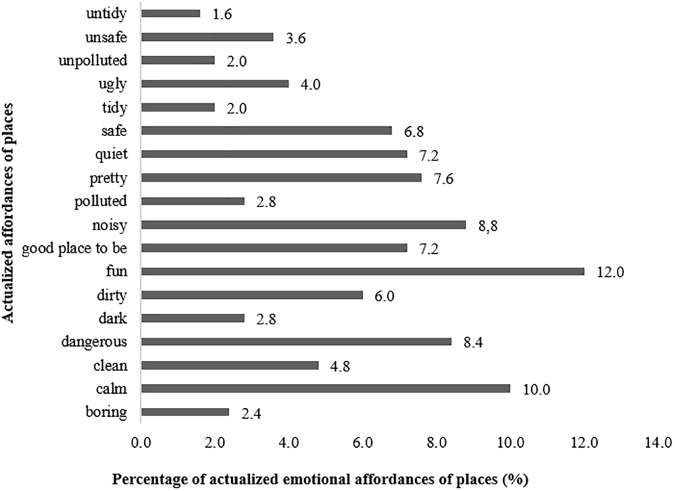
Percentage of emotional affordances of places actualized by participants in L group.

As for emotional type of affordances of places, the mostly expressive were “fun” (12%), “calm” (10%), “noisy” (8.8%), and “dangerous” (8.4%), as presented in Figure 7. The most expressive emotional sub-sets were “stressors” (34.4%) and “feelings” (31.6%).

### Neighborhood Area

#### Neighborhood Area and Actual Mobility to Affordances of Places

**FIGURE 8:**
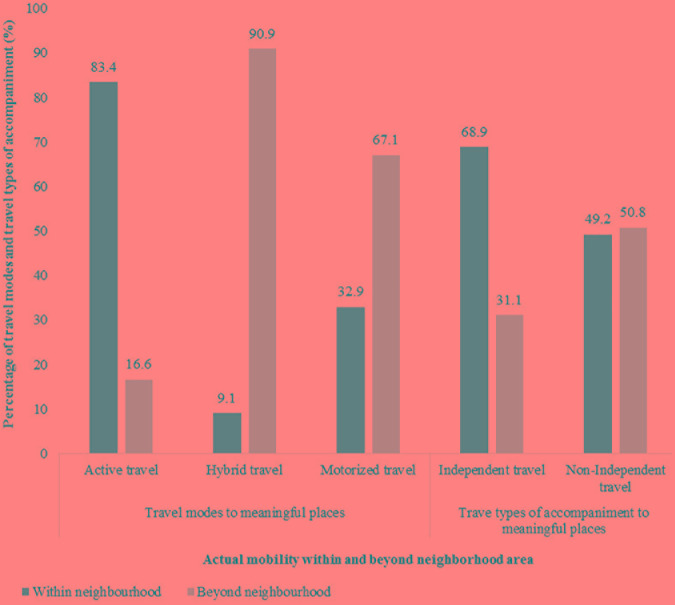
Descriptive interplay of mobility and territorial distance to meaningful places in L group.

Descriptive findings revealed an interplay between participants’ actual mobility and territorial distance covered by their traveling to meaningful places in the urban environment (Figure 8). More specifically, most active travel (83.4%) and independent travel (68.9%) occurs within neighborhood area (500 m buffer around participants’ home), whereas the majority of hybrid travel (90.9%) and most of motorized travel (67.1%) takes place beyond the neighborhood area.

#### Neighborhood Area and Expressional Categories of Affordances of Places

Considering the four expressional categories of affordances of places, descriptive results showed social affordances of places as the most expressive type (42.9%) within the neighborhood area (500 m around participants’ home). The values found for the other three categories are very similar, 18.6%, 19.6%, and 18.8%, for functional, leisure and emotional place types of affordances, respectively.

## Discussion

### The Interplay of Youth’s Mobility and Affordances of Places in the Urban Environment Toward Assessing Youth-Friendliness Degree of Neighborhood

In the Great Lisbon area, when returning home from school, an increasing trend of active and independent travel as children grow older is similar to evidence found in other studies (Davison et al., 2008; Fyhri and Hjorthol, 2009; Fyhri et al., 2011). In the present investigation, shorter school-home distances was pivotal in terms of active travel promotion. Participants’ active travel from school to home takes place if school-home mean distance is around 1.1 km. Although the threshold school-home distance for active travel is less than what it was found in a recent study which identified distances of 1.4 km for children at 10 years of age, 1.6 km at 11 years of age and 3 km at 14 years of age (Chillón et al., 2015). Our findings are similar to those found by Broberg and Sarjala (2015). More specifically, in the region of Helsinki, mean school-home distance was of 1.8 km; longer home-school distances decreased the likelihood of children and young people using active travel; and that within 1 km of school-home distance, majority of participants used active travel forms.

In terms of the school-home-journey, actions should be undertaken in order to increase levels of independent and active mobility for the younger ages.

In what concerns actual mobility to meaningful places in the Great Lisbon area, across all age groups active travel was the most frequently mode used to roam in the urban environment. It is interesting to notice that older children prefer hybrid travel mode to meaningful places, probably because it allows for them to move autonomously to further places, enhancing territorial range, and therefore mean distance traveling using hybrid mode was the highest one (3.3 km). Likewise, Broberg et al. (2013b), in a SoftGIS*children* study found that children aged 11 years old traveled significantly more often to meaningful places adopting active travel modes; whereas older children (aged 14) used public transportation and motorized car travel. In the present study, older children traveled autonomously more frequently to meaningful places and less often in company of adults than younger children did. On the contrary, Broberg et al. (2013a) found no significant differences in terms of type of accompaniment when reaching meaningful places between the younger and older group of children.

Our results revealed gender as an influential variable in terms of travel accompaniment to meaningful places. More frequently boys than girls traveled independently (by themselves or in company of friends) to these places; and more frequently girls than boys traveled accompanied by adults, which is in accordance with previous studies (e.g., Broberg et al., 2013a). Nevertheless, in the school-home journey, no significant differences were found on actual mobility between boys and girls. Our findings showed that only 44.3% of participants traveled autonomously from school to home, while 68.7% traveled independently to meaningful places, which possibly means that freedom for youths to roam independently in the school-home journey is more constricted and dictated by the adults’ agenda than when youths are running their own time. It is possible that parental fears and anxieties related with safety concerns and well-being are upheaved when children and youths have time and space to engage in contexts that afford their own agendas. In a study on gender differences and independent mobility of children aged 8–12 years old living in urbanized areas, Brown et al. (2008) found that boys were more likely than girls to travel alone to the park, sports facilities, cinema, shopping center and to the local shops. Conversely, girls were more likely to travel with an adult to go to all places and were less likely to move around within their surrounding home area. A more comprehensive approach revealed that for girls, the pathway for independent mobility emerges from the need for social networks of peers, use of public transport to reach semi privatized public spaces (as shopping centers) that are localized at a greater distance from home. On the other hand, for boys, independent mobility emerges from roaming freely in the local area and from interactions with the physical environment that afford physical activity play. Therefore, it is possible that the gender differences we found in our research, where boys traveled more frequently alone to meaningful places than girls did, with the exception of the school-home journey, where no gender differences were found, not only relate to a greater parental protectiveness over girls (when they are running own agendas) but also to a different co-productive emerging pattern of independent mobility.

Our findings on mobility to meaningful affordances of places linked with previous results from other research lead us to conclude that it is important for local muncipalities to raise existing moderate levels of independent and active mobility for both boys and girls, and increase their territorial range, by making enviornments safer and less hostile for children and youth. In a study focused on children’s independent mobility in three distinct urbanized environments (city, small town, and village), Lopes et al. (2014) have found that the percentage of Portuguese children living in the city of Lisbon who are granted freedom and autonomy to roam independently increases with age. In this same study, parents refer traffic danger and danger from adults as the main fears to grant autonomy of movement to children. Also, the influences of gender in parental fears regarding children’s safety were only relevant in the city environment, where danger from adults was the most representative reason for parents to pick their daughters up from school. We believe that an increase on territorial range may invite youths to engage in a more diversified range of place experiences, which would contribute for more time spent in active and independent travel modes. Previous research has shown that active and independent travel mode has been linked to good health and well-being of children and youths (Mackett et al., 2005; Mackett, 2013). Therefore, we believe that an effort of municipalities to increase youth’ territorial range could be perspective as health and well-being promoting measures within communities. Also distance from home to meaningful places must be considered when planning healthy and active environments for children and youths. In this sense we agree with Broberg (2015) that distance should always be considered when analyzing the relation between mobility, built environment characteristics and actualization of affordances. When comparing Portuguese children’s independent mobility with the Northern European reality, namely with Finnish children and youth (Kyttä et al., 2012), Portuguese standards are still low, namely for younger children. Moreover, in a recent international comparative study conducted on 16 countries, children’s independent mobility in Finland was found to be the highest (Shaw et al., 2015), whereas Portugal shares with Italy the 14th rank position. Also, our findings comparing real and ideal mobility, disclose that the vast majority of children would like to be more active, decrease car chauffeuring, and be more autonomous when traveling in the urban environment. Correspondingly, in Italy, at children’s councils and planning participation sessions, children have reported a desire for playful cities that enable free roaming and bodily autonomy (Tonucci and Rissotto, 2001).

The participants of this study identified a total of 1777 meaningful places. Mean number of meaningful places by participant was of 12.26. Former research conducted by Kyttä et al. (2012) and Broberg et al. (2013b), both in larger sets of participants, adopted SoftGIS methodology, and obtained a mean number of meaningful places per participant of 7 and 6, respectively. The differences concerning number of actualized affordances of places located by participants using SoftGISchildren surveys between our findings and those found in Finland may derive from the some specificities of the data collection procedure. In the Portuguese case, as mentioned in Section “Methods and Data Collection,” number of participants was much smaller (145) and each data collection sessions occurred with 10 participants and the co-presence of the researcher, or, when the number of participants was higher than 10, with the researcher and a research assistant. The role of the researcher and of the research assistant was to facilitate participants’ use of the SoftGISchildren survey. Specifically, before participants were allowed to start responding to the questionnaire, the researcher presented an overview of the survey by projecting it to participants on the interactive board. The researcher browsed the survey from the first to the last questions, testing it and explaining how the SoftGIS application should be used in order to provide answers. Moreover, when participants were completing the survey, at any time, they could call the researcher or the research assistant to clarify any doubts concerning any question on the web-map questionnaire, or to help them to overcome obstacles, namely, and most frequently, zooming areas and using commands, buttons and props to select, locate and save affordances of places, when producing their answers. It is possible that the role of the researcher and the research assistant as facilitators of the participants’ performance, when filling out the Ideal City SoftGISchildren survey, may have encouraged users to provide a more complete perspective of their psychological and spatial narratives of daily life in the urban environment. This was translated by an increase on the number of affordances of places identified in our investigation, when compared with those obtained by the Finnish studies.

In the present study, participants marked more social affordances of places, followed by leisure, functional and emotional types. Functional affordances of places, which are intertwined with physical activity play, were least frequent, possibly because our research participants are pre-adolescents and adolescents. Teenage behavior is very much characterized by social interaction among peers and social isolation (Clark and Uzzell, 2002) and by a decrease in physical activity play (Pellegrini and Smith, 1998). Nevertheless, our findings contrast with those by Sarjala et al. (2015) where most affordances of places marked by 5th and 8th graders were considered functional, tailed by emotional and social ones. The differences between our research findings concerning the expressive categories of place transactions (social, functional, leisure, and emotional) and the findings on the study conducted by Sarjala and colleagues may result from a combination of cultural factors. These relate to parental control and permission to engage in specific activities or behaviors; and simultaneously the type of features, elements and properties the immediate physical settings and the urban built environment offers youths in terms of actualized affordances. In a previous work (Lopes and Neto, 2014), the concept of “*Playgroundian City”* has been described as an urban environment which offers their citizens, including, children and youths, a transactional richness where actualization, shaping, reshaping and emergence of play, leisure, social, and emotional affordances are available across different urban spaces. It may be that the Finnish urban environment, due to urban planning and a conscious and deliberate design, offers more possibilities for youths to engage in functional affordances, and thus physical activity play and motor behaviors; as it does in what concerns opportunities for independent mobility, as it is clearly demonstrated by the Finnish rank position (1st) and the Portuguese one (14th) in an international study involving 16 countries on children’s independent mobility (Shaw et al., 2015).

In our investigation, within social categories of affordances, those with a higher expression of actualization were “being with friends,” “being myself,” “being with adults,” “being with animals,” and “being in peace and quiet.” Some of these findings are similar to those presented in a seminal SoftGISchildren research in the city of Turku, in Finland, conducted by Kyttä et al. (2012) with 1387 participants aged between 10 and 15 years old. These researchers found that most frequent social affordances were “meeting with friends,” “being yourself,” and “being in peace and quiet.” Generally, when comparing actualized meaningful affordances of places found in our research, in the Great Lisbon Area, and those from the city of Turku, it seems that social, functional and leisure experiences of Portuguese and Finnish children and youth are transversal in spite of country cultural specificities. In the present study, the innovative grouping of social affordances of places revealed that youths selected, more often, social places that allowed for “relational” and “affectivity” transactional experiences. The prevalence of the “relational” cluster reinforce Clark and Uzzell (2002) findings on neighborhood, school and town center as contexts that promote social interactivity and social withdrawal. Moreover, social interactions in the home, school and neighborhood environments are fundamental for the development of place identity and learning of social roles (Proshansky et al., 1983).

In the present study, the type of relational and affective immediate place experience and meanings that neighborhood area was found to afford youth are, in our perspective, key emotional features for place attachment to take place at the neighborhood level. According to Raymond et al. (2010), the attributes of the physical and social settings, which are personal contexts of place attachment, need to be included in the formation of individualized emotions in those same settings. In this way, social affordances of places that are actualized at the neighborhood level seem to be relevant for an *emotionalization* of place attachment. Kyttä (2003) refers to *emotionalization* as pivotal for the actualization of an affordance to take place and that every affordance has its own person-based emotionality fit. This means that the motivational basis for an actualization of an affordance relates to its *emotionalization.* According to Stedman (2008), settings have the potential for users to create multiple embodied cognitive meanings, referring to the descriptive symbolic meaning that people attribute to a place (Stedman, 2016). Our results on the neighborhood as a promoter of social affordances show that youths value this socio-physical context because it provides them with meaningful opportunities to be themselves, relate to each other and establish friendships and to be in peace and quiet. Some of the affordances of places that are most expressive for youths may be more related with place use, while others may refer more to place meaning. Either way, it seems that place use and place meaning, although they are different concepts, may be perceived directly and immediately by the person when actualizing a place-affordance. Therefore, the *emotionalization* of affordances of places also plays a role in terms of place meaning. In this way, the meaning of an affordance also resides on the intensity of the emotionality associated with its expressional multi-dimensions (functional, social, leisure, emotional, etc.) in consonance with the environment multidimensional character. Through the use of the hypothetical taxonomy adopted to categorize social affordances of places, it was found that the most expressive sub-categories which were actualized in the neighborhood were of relational and affectivity types of affordances. Therefore, we propose that the urban neighborhood enables youth to detect relational and affectional properties that are emotionally rich and meaningful.

### Youth-Friendliness Degree of Neighborhoods: From Space to Place

In the present study, active and independent travel occurs mostly within neighborhood area (500 m around participants’ home), whereas the majority of hybrid travel and most of motorized travel (takes place beyond this radius). Also, our results found neighborhood area as most prevalent in social affordances of places, since nearly half of them, within the four expressional categories, were actualized there. To assess the degree of youth-friendliness of neighborhoods, we used the hypothetical model proposed by Kyttä (2004). Here in, environments characterized with high levels of independent mobility and diversity of actualized affordances, where one correlates with the other, were designated as “*Bullerby”* and considered “child-friendly.” Also, a more recent work (Broberg et al., 2013a) on assessing child-friendliness of places was included, where place experience and place meaning contribute to the quality of child-friendly places. In this sense, it is more realistic to refer to child or youth friendly places than environments. Based on the above, in our study, the neighborhood area is proposed as meaningful setting for social interaction and for independent and active travel of children and young people, whereas beyond neighborhood areas seem more capacitated to promote functional, leisure and emotional affordances of places, and hybrid and motorized travel. It is very positive that within these 500 m, children largely enjoy independent and active mobility to meaningful places where a high frequency of social affordances are perceived and actualized. Independent and active mobility has been associated with children’s health and well-being (Mackett and Paskins, 2008; Fagerholm and Broberg, 2011) and as a correlate for physical activity (Schoeppe et al., 2013). In a systematic review, Sallis et al. (2000) found that time spent outdoors is consistently and positively associated with physical activity of children and adolescents. Moreover, in pre-adolescence and adolescence, youths are attuned with social activity as part of an internal and external social construction of childhood and place identity. It is therefore possible that when youths perceive and actualize affordances of places in the neighborhood area, the mutuality between the person and the environment is leading to a more socially dominant demeanor, thus making the “eco-niche” more socially meaningful. The eco-niche emerges from the interaction of the information that specifies the functions of the environment with the information that specifies the corporal aspects of the person (Gibson, 1979/1986). Previous research concluded on the importance of the 500 m socio-built environment around young people’s homes as a fundamental promoter of the neighborhood’s free roaming (Fagerholm and Broberg, 2011); and another study reinforced the importance of the neighborhood as a promoter of youths social retreat behaviors (Clark and Uzzell, 2002). Also, and considering that within social affordances of places those more frequently actualized were “being with friends” and “being myself,” such finding concurs with one presented in a previous research on children’s independent mobility and degree of urbanization conducted in Portugal (Lopes et al., 2014). More specifically, it was found that within activity places children traveled to independently on their leisure time, “going to a friend’s home” was among those mostly reported. Hence, in the present study, neighborhood areas were assessed as youth-friendly because they were found to be socially meaningful *bullerby* types of settings.

Concerning the production of places, Tuan (1983) sustains that abstract space becomes place as it is progressively experimented and practiced in daily life. Likewise, in a study about how girls create meaning of place through collective autonomy, Christensen and Mikkelsen (2013) synthetize ideas of previous scholars about place meaning sustaining that space becomes cultural and embedded of material, social and symbolic meanings through bodily presence and active co-participation in their immediate surroundings; through this process children create their own sense of place, dialogically and negotiating with others their material, social and cultural worlds. Moreover, neighborhoods are referred to as embodied socio-cultural ecosystems (Raymond et al., 2017a) nested in specific affordances and specific places (Heft, 2018). Drawing on the above, we suggest that recurrent daily actualization of affordances of places and free roaming over time through the neighborhood enable children and youths, collectively and progressively, to actively participate in the co-emergence of multi-dimensional place meanings, through an entanglement of body, mind, environment and culture, turning spaces to meaningful places. On this, Heft (2018) sustains that to understand human perception-action at a communal level it is indispensable to recognize that actions in the environment are nested to the affordances of objects and to the affordances of place, which emerge through social participation. The combination of autonomous corporeal movement and creation of multidimensional meanings of place through active place participation across the neighborhood allows youths to become *specialists of space*, or *spatialists*. Moreover, the use of SoftGISchildren surveys allows participants to inform about such place meanings and daily psycho-spatial narratives, which reinforces the perspective of youths as *spatialists*, in the sense of informing about their embodied *spatialism*.

### Research Limitations and Future Investigation

We believe this research to be noteworthy as it provides a valuable and innovative insight to address the interplay of mobility, affordances of places and the neighborhood area. However, some limitations should be considered. Firstly, participants were asked to select affordances of places and localize them on the web map in the place where these interactions occurred. This means that some of the places which were marked on the web map may have been located in a nearby area, without really considering the place which was intended to. Consequently, it is possible to have existed place discrepancies between real intentioned place and the digital perspective of places. Nevertheless, and due to the nature of the research work and to the data collection instrument that was used, these limitations could not have been overcome. In spite of these setbacks, 1777 meaningful places were located. Secondly, the grouping of affordances on each of the expressional categories (social, functional, leisure, and emotional) was empirically driven and devised based on criteria defined by the author. Future studies should test these subsets of affordances using appropriate statistical based clustering. Likewise, other criteria for each category of affordances could have been selected. However, each of the four criteria was coherently justified and applied within those terms. Thirdly, most statistical analyzes used in this research were based on descriptive statistics and inferential statistics were just used when analyzing specific relations between variables. Although we believe that for the exploratory-descriptive kind of study this approach proved to be effective, we do realize that certain data assumptions which were made must be read bearing this statistical limitation.

A careful consideration related with the use of linear distances has to be made when interpreting our findings on the interplay of distance between home and school and mobility behaviors and between home and meaningful places; and on the interplay of distance and actualization of affordances of places, namely, when considering the 500 m neighborhood buffer. Linear distances do not correspond to the real distances traveled by youths, adopting any type of travel mode or of travel accompaniment, from home to school and from home to other meaningful places. Whichever ways youths use to move around, traveling is conducted using the street network and thus distance is not linear. A better measurement would have been to adopt the cognitive distance related to the street networks using space syntax or place syntax tools. Nevertheless, the use of linear distances, the shorter path between two points (crow-flies) has been adopted in other studies on active living research with children and youths, such as Broberg et al. (2013b) and Broberg and Sarjala (2015) when considering distance between home and meaningful places. As for the use of 500 m radius buffer around youths’ home, the same procedure was applied by Kyttä et al. (2012) in a seminal research using SoftGISchildren methodology. In spite of the limitations, our findings, although just indicative, are a valuable contribution for municipalities when planning and designing youth-friendly ecosystems, such as the neighborhood area. Linear distances applied to the context of independent mobility and affordances may be very useful for municipalities to have access to the territorial boundaries of youths’ spatial, place based narratives. This type of knowledge is important to implement measures that promote the extension and the enrichment of free roaming meaningful transactional territory for youths. Future studies on this topic should address this issue adopting an approach based on street networks and connectivity. Nevertheless, the approach to distances between places adopted in the present research provides an important contribution for municipalities to design and manage youth-friendly ecosystems that promote well-being and contribute to a sustainable development.

In spite of these limitations, this study contemplates innovative aspects and offers interesting possibilities for future research in Environmental Psychology. To the best of our knowledge, this is the first investigation conducted in Portugal using SoftGIS*children* methodology to study children’s independent mobility and child-place interactions in the environment; and, internationally, the first time where grouping of affordances within expressional categories was implemented. Future research, should explore grouping of affordances of places, validate, or redefine the used taxonomy for different expressional categories of affordances of places. Studies using SoftGISchildren methodology have focused on children’s daily use of places, however, and as far as what we are concerned, less attention has been paid to how children’s and youths’ perspectives on how they would like their urban environment to be. Our research through the analyses of youth’s actual mobility vs. ideal mobility to meaningful places provides a starting and seminal point to address these concerns using SoftGISchildren methodology. Also, in our opinion, future studies using SoftGISchildren methodology should include children and youths as co-designers of the web-map place based enquiries by integrating functionalities in the web-map survey to allow participants to graphically imagine child-friendly structures and settings. Moreover, SoftGIS*children* methodology should be combined with other data collection methods, such as, semi-structured interviews; mobility diaries, GPS data, accelerometers, photo-voice; neighborhood walking; etc. A very interesting example of this was the development of an application called *Mappiness* used in a study conducted in the United Kingdom. At random moments, participants were signaled through the app to answer to a small survey while their exact spatial location was recorded using GPS coordinates (MacKerron and Mourato, 2013).

### Practical Implications

In an ever growing urbanized world and considering the recent agenda for sustainable development goals (United Nations, 2015), planning, designing, and redesigning urban landscapes toward the promotion of health and well-being of children and youths constitutes an immense challenge for governments, municipalities, policy makers and urban planners. In fact, it is concerning that over the past 50 years, there has been a decline of children’s outdoor free play with other children, accompanied with the rise of psychopathologies in this population (Gray, 2011). Also, there has been a decrease of independent mobility and of social relations with others, which minimizes the access and use of residential streets in neighborhoods by children and youths for leisure, recreation and play activities (Shaw et al., 2015; Tranter, 2015). This is aggravated by an increase of overweight and obesity and decrease of physical activity among children (Whitzman et al., 2010). Hence, it is crucial to identify a set of practical implications that yield from the present study to tackle these problems.

#### Affordances of Places as Multidimensional Concepts

Affordances of places captured by place-based surveys offer the possibility for using them when planning environments that afford iteratively reconfigurations. When planning a neighborhood is important to create settings that are open-ended, where meaning of place results from diversified activities, feelings, values and uses, which also play a key role in the development of sense of place and identity.

#### Beyond Urban Neighborhoods That Privilege the Actualization of Social Affordances of Places

The findings of our study revealed the neighborhood area as most prevalent in social affordances of places. Although this represents a positive finding in line with the developmental needs of the study’s participants (pre-adolescents and adolescents), we believe that neighborhoods should also be designed to youths with interesting, intriguing and challenging play, leisure and recreational opportunities.

#### Free Roaming of Youths as a Crucial Aspect of Daily Life in Urban Neighborhoods

Urban neighborhoods should be planned with places and routes that afford independent and active mobility of children and youths, which is associated with physical activity, health and well-being. This type of planning also promotes a diversified place experience of the territory.

#### Youths as Actors, Participants, and Co-designers of Planning Practices

SoftGISchildren methodology enables the empowerment of children and youths as active-participants by providing relevant information to planners about their perceptions and experiences in the urban spheres, by digitally detecting affordances of places and reporting actual *versus* ideal mobility to meaningful places.

## Conclusion

The use of SoftGIS real-ideal survey mapping has proved to be an effective youth-friendly process that enables participants to digitally report about their immediate place experiences and place meanings as real life actors that simultaneously influence and are influenced by the close environment. This perceptual communality between the physical and the digital place experience and meaning stresses place-based mapping as a more ecological methodology than the use of more traditional methods such as interviews, questionnaires and diaries. It is important to address the neighborhood area as a youth-friendly embodied ecosystem that promotes free roaming and social meaningful places and the claim of increased autonomy of movement by young people. Also, children and young people ought to be considered as pivotal actors and providers of meaningful information for urban planning processes. Public polices to promote youngsters’ health, well-being and happiness should therefore include active processes of participation, where the real and ideal city is critically discussed by youths.

## Author Contributions

FL was the project leader and the corresponding author. FL did all of the field-work and did most of the writing with the contributions of RC. CN participated by discussing theoretical assumptions with FL and with RC.

## Conflict of Interest Statement

The authors declare that the research was conducted in the absence of any commercial or financial relationships that could be construed as a potential conflict of interest.

## Footnotes

1 • Single choice questions concerning actual and ideal travel mode from school to home were asked. Answers had to be selected from the following options, on foot; by bicycle; by bus/by public transport; by car; by other (skate, scooter, roller-skate, etc.). Single choice question concerning daily and ideal travel accompaniment from school to home were asked. Answers had to be selected from the following options (alone; with other children; with adults; with adults and other children).

• In what refers to affordances of places, after selecting one and locating it on the web-map, participants were asked about actual and ideal travel accompaniment to meaningful place where the affordance was located. For each one, multiple choice answers could be given. “I travel to this place” (actual) and “I would like to travel to this place” (ideal) according the following options, alone; with other children; with adults; with adults and other children. This procedure was repeated each time a place-affordance of any considered category (social, functional, leisure, or emotional) was located.

2 Bellow, a more detailed explanation on the procedure for grouping the affordances in each expressional category is presented:

• A common denominator for all affordances of places which was “place interactions where” (as it is displayed in Tables 1–4) was designated by the authors.
• For the social, leisure and emotional expressional categories, affordances were grouped based on possible communalities content wise, on a trial and error basis. This enabled to stance out a specific, however, subjective, criteria between those affordances that shared similarities for each expressional category of affordances of places. (i.e., “it is mainly valued being alone and free from the public eye,” “it is mainly valued being connected with others”…).
• For the social, leisure and emotional categories of affordances, each common denominator that emerged (sub-set) and that specify each criteria in Tables 1, 3, 4 was renamed according to a broader concept for each sub-set, which was also defined by the authors’ subjective point of view (i.e., in social category, the proposed sub-sets were privacy, relation, license, affectivity).
• The “Oxford University, 2015” source was only consulted afterwards in order to reassure that the match between each specific criteria and each sub-set was possible. This was also a subjective analysis conducted by the authors.
• For the functional category of affordances, grouping of affordances was based on possible communalities content wise guided by the play types defined in Bob Hughes play taxonomy. The affordances were subjectively grouped but considering a possible inclusion of each action within with each considered play type. The criteria which was used is the definition of each considered play type and the sub-set is the designation of the play type itself.
